# Optical metasurfaces: new generation building blocks for multi-functional optics

**DOI:** 10.1038/s41377-018-0058-1

**Published:** 2018-08-29

**Authors:** Dragomir Neshev, Igor Aharonovich

**Affiliations:** 1 Nonlinear Physics Centre, Research School of Physics and Engineering, Australian National University, Canberra, ACT 2601 Australia; 2 Institute of Biomedical Materials and Devices, University of Technology Sydney, Ultimo, NSW 2007 Australia

## Abstract

Optical metasurfaces (OMs) have emerged as promising candidates to solve the bottleneck of bulky optical elements. OMs offer a fundamentally new method of light manipulation based on scattering from resonant nanostructures rather than conventional refraction and propagation, thus offering efficient phase, polarization, and emission control. This perspective highlights state of the art OMs and provides a roadmap for future applications, including active generation, manipulation and detection of light for quantum technologies, holography and sensing.

## Optics: Metasurfaces bring new opportunities to light

Materials with ‘optical metasurfaces’ (OMs) have surface layers patterned at scales smaller than the wavelength of light, offering fundamentally new ways to manipulate light for many possible applications. Conventional optics generally relies on light being refracted as it passes through materials, or simply reflected from surfaces. Igor Aharonovich at the University of Technology Sydney, and Dragomir Neshev at the Australian National University, Canberra, review the current capabilities and likely future applications of OMs. The authors themselves investigate light capture, scattering and re-emission by OMs to control aspects of light including its phase, polarization and emission characteristics. They foresee new ways to control the properties of light serving to advance technologies including those that exploit the subtle quantum mechanical properties of light. Innovative optical sensors and holography systems are among the many potential uses of OMs.

## Introduction

**Fig. 1:**
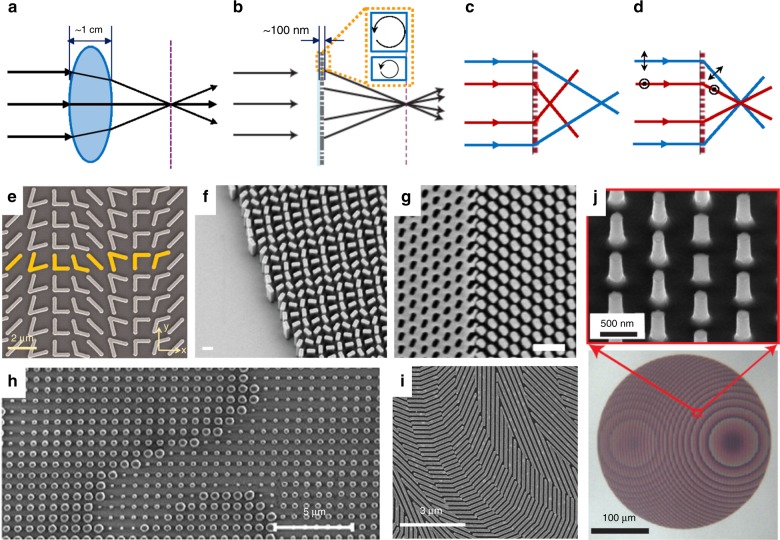
**Optical Metasurfaces.** **a** Conventional optics (lens) relies on refraction to bend light beams, while a metasurface (**b**) bends light due to scattering by nanoparticles of different sizes. **c**, **d** Multiplexed control of different colors and polarizations by a metasurface. **e** OM composed of a gold antenna array. The unit cell of the plasmonic interface (yellow) comprises eight gold V-antennas^3^. **f** A metalens operating at 660 nm and consisting of TiO_2_ nanofins on a glass substrate. Scale bar is 300 nm^7^. **g** Achromatic metalens with NA ~0.1. Scale bar is 500 nm. The vertical boundary of nanopillars and Babinet structures is visible^8^. **h** Fabricated meta-hologram that produces 5 mm large images at a distance of 10 mm. The posts are silicon on SiO_2_^9^. **i** SEM image of a dielectric metasurface lens based on Si nanobeams that results in a local Bessel spot focal length of 100 mm at λ = 550 nm^10^. **j** DM made from amorphous silicon pillars on a SiO_2_ that separates *x*- and *y*-polarized light and focuses them to two different points^11^

Optical metasurfaces (OMs) are sub-wavelength patterned layers that interact strongly with light, thus dramatically altering the light properties over a subwavelength thickness. In contrast to conventional optics, which rely on light refraction and propagation (Fig. 1a), OMs offer a fundamentally new method of light manipulation based on scattering from small nanostructures (Fig. 1b). Such nanostructures can resonantly capture the light and re-emit it with a defined phase, polarization, modality and spectrum, thus allowing the sculpting of light waves with unprecedented accuracy. This ability to manipulate light at the nanoscale level has opened a plethora of practical applications, including spectral selectivity (Fig. 1c), wavefront and polarization control (Fig. 1d), and the control of light radiation and detection. OMs have strong similarities to frequency selective surfaces and high-contrast gratings^1^; however, they have experienced enormous advances in their complexity and functionalities over the past decade.

Initially, OMs were composed of metallic (plasmonic) nanostructures, following the advances of the field of metamaterials and drawing on subwavelength light confinement in such nanostructures. The first OMs were generated using periodic or distributed arrays of resonant gold antenna arrays (see Fig. 1e) on silicon oxide and were used to “bend” light beams^2,3^ by phase manipulation. These first results inspired scientists to create ultra-thin optical elements, opening the field of metasurfaces^3,4^. However, due to the strong Joule losses in the metallic nanostructures composing the metasurface, plasmonic OMs have hit a fundamental limit to their performance and efficiency. An important alternative has quickly emerged in the use of dielectric nanoparticles as nanoscale building blocks. Dielectric nanoparticles exhibit strong Mie-type resonances of both electric and magnetic nature^5^, where the resonant wavelength is proportional to the size of the particle multiplied by the refractive index of the dielectric material. At resonance, the particles have induced-electric or magnetic dipole (or higher-order) moments, the interference of which strongly affects the directionality of the scattering. For example, at the spectral overlap of the electric and magnetic dipoles, the nanoparticle scattering is unidirectional in forward directions. Therefore, the transmission through an OM of such nanoparticles is unitary, while the transmitted phase can vary in the full range, 0–2π^6^. This Huygens-type regime has opened the application of dielectric metasurfaces (DMs) for transmissive-type optical devices with an efficiency that is approximately unitary.

The fabrication of DMs has relied on a number of high-refractive index dielectric materials, which offer different opportunities and challenges. The constituent nanostructures of DMs (see Fig. 1b–f) have sub-wavelength dimensions, which require the use of advanced nanofabrication techniques (e.g., electron beam lithography). The material of choice for DMs has largely been silicon (Fig. 1e–j), which offers compatibility with complementary metal-oxide-semiconductor fabrication, thus merging electronic chip-making with optical design and fabrication. The drawback of using silicon is its low transparency in the visible spectral range, requiring other high-index materials that are transparent in the visible range.

### Current applications of metasurfaces

Over the past few years, the field of OMs has rapidly expanded to include a myriad of future applications in the field. The important development milestones of flat meta-optics have been the demonstrations of metalenses^7,8^, beam-shaping devices^10^, polarization optics^11^, and holograms^9^ (see examples in Fig. 1). Below we provide a short overview of these meta-optics components.

#### Metalenses

Metalenses are among the most commonly explored meta-optical elements. Their development has been driven by applications in microscopy and imaging. The initial results have been focused on low numerical aperture (NA) lenses at infrared wavelengths. However, improvements in design have allowed for high NA (>0.9) metalenses, opening the way for compact imaging microscopy systems. Metalenses fabricated from wide bandgap transparent materials, such as titanium oxide^7^ and gallium nitride^12,13^, have been the solution to improving the performance in the visible spectral range. Monochromatic aberrations (such as coma and astigmatism) have also been addressed using metalens doublets^14^. While these metalens devices have shown good performances, they only operate at a single wavelength. Broadband operation of metalenses has been one of the biggest obstacles in the field, requiring the development of new designs for compensation of the chromatic dispersion. Recently, this barrier was overcome using a special design of the geometric dispersion of the metasurfaces, resulting in a demonstration of broadband achromatic metalenses in the visible range^8,15^, paving the way for applications in the smart-phone and display markets.

#### Meta-holograms

Designing holograms often requires the encoding of a complex phase distribution and multiple bulky elements (lenses/splitters and sometimes more than one light source). The concept of metasurfaces allows for the planar fabrication of such complex phase distributions in a single layer, without the need for complex multi-layer lithography. Importantly, both the phase-only and the amplitude and phase complex phase distributions can be achieved. Some of the first meta-holograms were realized in reflection geometry, using plasmonic elements on a “ground” (mirror) plane. Complex holographic images were reconstructed with efficiencies up to 80%^16^. The use of DMs has further allowed for meta-holograms working in transmission and reaching efficiencies of over 90%^9,11^. To date, most meta-holograms have been limited to a single color (i.e., grayscale); however, by interleaving multiple holograms on a single surface, it is possible to realize multicolor holograms^17^. The challenge with current meta-holograms is their “permanent” display since the pattern cannot be changed after fabrication. This stimulates the development of reconfigurable meta-holograms that are yet to be realized.

#### Polarization optics

Polarization is one of the most important properties of light beams and can be controlled using birefringent or chiral materials. OMs are unique in regard to the possibility of creating the strongest artificial birefringence. For example, OMs composed of subwavelength elliptical posts of a high-refractive index material can be used to realize waveplates or q-plates for the generation of vector beams with a complex polarization. The ellipticity of the posts result in two effective refractive indices for the two orthogonal polarizations, which creates an artificial birefringence and controls the output polarization of the beam. By varying the orientation of the posts across the OM, one can encode a spatially variant birefringence in a selective fashion^11^, with high transmission efficiencies of up to 97% over a wide spectral band.

### The new frontier—active metasurfaces

**Fig. 2:**
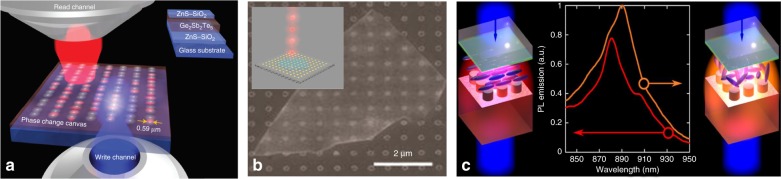
**Active metasurfaces.** **a** Schematic illustration of reconfigurable photonic devices in a phase-change film with optical components, such as lenses and resonant metamaterials, written in a chalcogenide glass phase-change film using fs pulses. The written pattern can also be erased using the same laser with different illumination conditions. The results are observed through the “read” channel^18^. **b** Example of a 2D material, layered hBN, positioned on a plasmonic metal array for achieving enhanced single-photon emission. The inset is the second-order correlation function that confirms that a single quantum emitter is probed^20^. **c** Schematic illustration of a DM integrated into a liquid crystal cell. By heating the liquid crystal, it changes its state from nematic to isotropic, resulting in a spectral shift of the metasurface resonances and the tuning of the emission enhancement^21^

Many applications such as holographic projections, beam steering for LIDAR or satellite quantum communications require fully active meta-optics, which can be dynamically reconfigured as well as used to generate or detect light. The development of active metasurfaces represents a new wave of research in the field; however, it also comes with many challenges, including the reconfigurability and generation of quantum and classical light, as shown in Fig. 2a–d.

#### Reconfigurable OMs

Active control of the metasurfaces, for instance by applying a voltage or strain, can result in fast structural modulation, thus yielding advanced color control suitable for camouflaging, multiplexing, multicolor holograms^18^ (Fig. 2a) or tunable focal-length lenses^19^. Similarly, embedding these metasurfaces into microfluidic channels to be deployed as high-resolution sensors is also appealing, due to their typical robustness and the relative ease of surface functionalization. A particularly fascinating application of OMs that encompasses the aforementioned progress can be envisioned within the emerging technology of transparent information displays for self-driving vehicles. The current challenge in the field is to achieve a phase-only tunability over the entire 2π phase range, which would open the way for dynamic beam steering for navigation for autonomous vehicles or dynamic holographic displays. Another paradigm shift is the realization of an active window that will not only project external information (e.g., speed) but also detect these rapid changes. This application will require sensor integration with highly advanced holography imaging.

#### Quantum metasurfaces

Current techniques for light enhancement often include lossy plasmonic elements or complicated structures of photonic crystals or cavities. The immediate applications in the field of quantum metasurfaces should include integration with quantum emitters, which will enable on chip, high-efficiency collection enhancement and directionality of emitted photons. This technology would be particularly relevant for emerging sources embedded in atomically thin 2D crystals, such as hexagonal boron nitride (hBN) and transition metal dichalcogenides (TMDCs). Initial experiments of light enhancement from a single emitter using an OM is shown in Fig. 2b^20^. Further experiments of coupling emitters to dielectric nano-cavities and exploring fundamental phenomena, such as Purcell enhancement, are also looming. Finally, the strong light confinement available with DMs can be leveraged to engineer new quantum multi-photon interferences at the subwavelength scale, which is currently not possible with conventional techniques. Capturing additional degrees of freedom associated with spatially varying polarization states can be further harnessed for free space quantum communication and measurement of the quantum state of light.

#### Emitting and lasing metasurfaces

OMs that can strongly enhance the atomic emission at the position of their resonance can lead to ultra-bright and directional light sources. These sources can be spectrally tuned by reconfiguring the metasurface (Fig. 2b)^21^. Work is currently underway to realize DMs based on GaAs pillars with embedded QDs that can be an effective medium for a laser^22^. These are adequate alternatives to rather complicated photonic crystal designs for achieving similar powers/thresholds. Developing lasers in the visible and, potentially, UV spectral range is appealing. The quality factors achieved with DMs are increasing, and with a suitable gain material, coherent lasing with complex-shape beams emitted from metasurfaces should be possible.

### Future challenges and opportunities

Currently, the majority of efforts have been dedicated to engineer a single optical functionality on a single layer of OMs. The metasurfaces are discrete optical components arranged in arrays of nanostructures, thus offering a unique opportunity to interleave multiple functions in a single layer^23^, which is not possible with conventional optics. For example, this technology may be used to achieve spin-dependent metasurfaces and to control the phase of a quantum emission to make quantum circuitry more practical. This is where new approaches for OMs design, incorporating symmetry, topology and disorder need to be developed. Precise design tools are also required to improve the current Q factors of DM. Finally, multiple metasurfaces can be vertically stacked using precise nanoscale alignment, scaling up of the number of devices on a chip from tens to thousands. Adding surface functionalization on top of the OM array can also yield ultra-sensitive, chemically specific techniques for versatile bio-sensing, avoiding the complex instrumentation currently required^24^.

The development of low-cost and large-area fabrication techniques is also needed to avoid relying on currently expensive electron beam lithography. A promising avenue is to explore bottom up techniques, such as DNA self-assembly, that have been used to assemble plasmonic nanostructures, as well as fabrication on flexible substrates for integration with other devices. Effort should be dedicated to designing and engineering efficient OMs from other materials, such as diamond and silicon carbide. While those have a lower refractive index than silicon or gallium arsenide, these materials host quantum emitters, a facile method to engineer quantum elements with dielectric metasurfaces. Working with diamond or gallium nitride could also push resonances into the UV spectral range, a highly envisioned spectral range where plasmonics or dielectric cavities are challenging to engineers.

While it is unlikely that meta-optics systems are going to replace standard desktop optical microscopes, building an entire on-chip confocal microscope using meta-optics for remote imaging is plausible. On-chip multicolor holograms that can be simultaneously used as displays and sensors is certainly within reach with the current technologies. Our vision for the future development of the field can be exemplified by a single ultra-thin DM coupled to a 2D material that integrates multiple functions for light emission, manipulation, and detection, together with an electrical control. Our design is not limited to a monolayer of a single material and can thus be multicolor. The progress of such active meta-optics systems is steady and will be quickly accelerated with industry giants adopting the technology in a range of their display-based technologies.
